# Prevalence and Assessment of Malnutrition Risk among Hospitalized Children in Romania

**Published:** 2014-03

**Authors:** Oana Mărginean, Ana Maria Pitea, Septimiu Voidăzan, Claudiu Mărginean

**Affiliations:** University of Medicine and Pharmacy Tg.-Mures, Gheorghe Marinescu Street, 38 Tirgu Mures, Mures 540139, România

**Keywords:** Child, Malnutrition, Serum proteins, Romania

## Abstract

Malnutrition is a prevalent condition in hospitalized children. Our aims were to evaluate the nutritional state and to validate the STRONGkids risk assessment tool in a hospitalized paediatric population in Romania. This is a prospective single-centre study in a tertiary teaching hospital in Romania (May 2011-January 2012). We calculated the STRONGkids score and measured the children's height and weight. Standard deviation <-2 for weight-for-height and height-for-age was considered to indicate acute or chronic malnutrition respectively. Two hundred seventy-one children were included, with median age of 5.2 years and median hospital stay of 2.01 days. Prevalence of malnutrition and severe malnutrition was 37% and 15% respectively. Using the STRONGkids screening tool, 58% of the children were found at risk of malnutrition (24% were at high risk). The kappa coefficient of agreement between STRONGkids and WHO malnutrition classification was 0.61. When a low serum protein level was used in upgrading STRONGkids risk category, kappa increased significantly to 0.71 (p=0.001). A modified STRONGkids score, incorporating total serum protein levels, performs well in predicting malnutrition in hospitalized paediatric population in Romania.

## INTRODUCTION

Malnutrition in children results in impaired growth, development, poor health, and overall decreased wellbeing (1). Nutritional status is essential for the management of children admitted to hospital (2,3) and is directly associated with the duration of hospitalization and healthcare costs (2). While primary malnutrition caused by poor nutritional intake is more frequent in the underdeveloped countries and is directly linked to socioeconomic status (4), malnutrition is usually secondary to a variety of chronic diseases in more developed countries. Reported prevalence rates of paediatric malnutrition in developed countries vary between 6.1% of children in Germany (5), 6.9% in Brazil (6), 21% in France (7), and up to 31.8%, in Turkey (8,9). In hospitalized paediatric population, nearly half of the children with chronic diseases are malnourished (10-12). In Romania, the prevalence and risk of malnutrition in hospitalized children are not known.

Management and prevention of malnutrition in hospitalized children rely on early identification of those at risk in order to implement early nutritional interventions. For this purpose, several risk assessment tools have been developed (10,13-15). The STRONGkids screening tool is easy to use and has been validated in hospitalized children in several developing countries (16). The STRONGkids score is a measure of nutritional risk. The tool consists of 4 parameters (subjective clinical assessment−poor nutritional state; high risk disease—underlying disease with risk of malnutrition; nutritional intake and losses—diarrhoea, vomiting, decreased food intake; and weight loss or poor weight gain), each item receiving 1-2 point(s), with a maximum total of 5 points (13).

This nutritional score identifies three risk categories (low, medium, and high) and correlates well with WHO malnutrition standards (17). Its applicability to Romanian children has not yet been evaluated. In our study, we aimed to evaluate the validity of the STRONGkids nutritional risk assessment tool in a Romanian paediatric population admitted to a tertiary-level academic hospital.

## MATERIALS AND METHODS

Consecutive children admitted to Targu Mures University Hospital, a tertiary paediatric teaching hospital located in Targu Mures, Romania, were enrolled in this study between 1 May 2011 and 30 January 2012. We calculated the STRONGkids score and measured the height and weight of children admitted to the hospital; z-score [also called standard deviation (SD) score] quantifies the deviation of an individual's value from the median value of a reference population, divided by the standard deviation of the reference population (or transformed to normal distribution). We used this score to describe how far a measurement stands from the median (average) (18). Standard deviation of z-score <-2 for weight-for-height (WFH z-score) and height-for-age (HFA z-score) was considered to indicate acute and chronic malnutrition respectively.

Inclusion criteria were age between 1 and 17 year(s) and duration of hospitalization of at least 24 hours. Obese children (BMI at or above the 95^th^ percentile for children of the same age and sex) and those with active malignancy admitted during this time period were excluded.

The study was approved by the local Committee of Ethics of the University of Medicine and Pharmacy of Targu Mures. Informed consent was obtained from parents or legal tutors at the moment of admission to hospital in compliance with the principles of the Helsinki Declaration.

Baseline characteristics recorded from patient's medical record included age, gender, ethnicity, presence of an underlying chronic disease, admission diagnosis, and length of stay in the hospital (LOS).

### Anthropometric measurements

All measurements were made in a standard way by one operator (a paediatrician experienced in paediatric nutritional assessment) unrelated to the study. Weight in kg, and height in cm were measured and recorded for all children at the time of admission to the hospital. Body mass index was calculated as weight divided by square of height (kg/m^2^). Mid-upper arm-circumference (MUAC) in cm and tricipital skinfold (TSF) in cm were also recorded in the first 24 hours of hospital stay. Height and weight were measured according to standard procedures (19). Height was measured to the nearest 0.1 cm, using a wall-measured unit size. In children unable to stand, length was measured in cm while lying on a firm surface with a rigid headboard. Weight was measured to the nearest 0.1 kg, using a standard balance beam scale, with children barefoot and with hospital gowns only. MUAC was measured midway between the upper arm and the olecranon, with upper limb flexed at 90^0^, and the average of three measurements was used for analysis. TSF was measured midway between olecranon and humeral head by grasping a skinfold between the thumb and index, then placing a calliper; the average of three measurements was used for analysis.

### Assessment of nutritional state of children

The cohort was stratified according to WHO classification of malnutrition (17,20). Acute malnutrition or wasting was considered severe if WFH z-score was below −3 and moderate if between −2 and −3 SD. Chronic malnutrition or stunting was defined as severe when HFA z-score was below −3 SD and moderate when HFA z-score was between −2 and −3 SD. Serum levels of total proteins were measured spectrophotometrically on fasting blood samples on a Sebia microanalyzer (Evry, France), with a coefficient of variance of 0.1. Normal range for total proteins was 6.6-8.7 mg/dL.

### Statistical methods

Statistical analysis was performed using MedCalc Software [bvba (version 12.3.0), Mariakerke, Belgium] for a descriptive analysis of the population and the assessment of the malnutrition risk. Categorical variables were summarized as percentages and compared with Fisher's exact test for two groups and chi-square tests for three or more groups. Continuous variables were presented as medians with interquartile ranges and were compared using the Kruskal-Wallis non-parametric test or the Mann-Whitney two-sample test. Agreement between the STRONGkids and WHO malnutrition class was assessed with the kappa method of inter-rater agreement. A kappa level over 0.7 was considered clinically significantly. The significance level was set at p<0.05.

## RESULTS

Four hundred and fifteen children were hospitalized between 1 May 2011 and 30 January 2012 at our paediatric hospital; 326 children were found eligible for the study, of whom 271 agreed to participate. Baseline characteristics of the cohort are presented in Table 1; 150 children (55.40%) were male, and their median age was 5 years [range 1 to 17 year(s)]; 131 children (48.33%) had underlying diseases (chronic conditions) known to be associated with malnutrition: renal disease (n=10), cleft lip and/or palate (n=4), problems consequent to previous premature birth (n=8), bleeding disorders (n=18), chronic gastrointestinal disorders (n=30), neurodevelopmental disorders (n=19), respiratory conditions (n=22), cardiac malformations (n=13), and endocrine disorders (n=7). Diagnoses at admission included complications of gastrointestinal disorders (n=84), respiratory disorders (n=83), developmental abnormalities (n=28), immune-allergic disorders (n=17), haematologic disorders (n=16), acute surgical disorders (n=13), intoxications (n=10), neurologic disorders (n=4), infectious disorders (n=4), and renal disorders (n=3). Median (range) length of stay was 2.01 days (1-24); 63.1% were hospitalized for <3 days, and 15.1 % for >3 days.

The median (range) WFH z-score and HFA z-score were 0.1 (-4.5,3) and −0.34 (-7.6,8). The anthropometric parameters of the nutritional subgroups are presented in Table 2. As expected, median z-scores for all anthropometric parameters varied inversely with the degree of malnutrition.

**Table 1.** Demographic characteristics of the cohort

Characteristics	N=271
Median age in years (range)	5.2 (1-17)
Male gender, n (%)	150 (55.40%)
Median length of hospital stay in days (range)	2.01 (1-24)
Presence of underlying disease, n (%)	131 (48.33%)

**Table 2.** Anthropometric characteristics of the groups

Variable	WHO malnutrition class			STRONGkids		
	Low (n=171)	Moderate (n=59)	Severe (n=41)	Low (n=115)	Moderate (n=92)	Severe (n=64)
WFA z-score (range)	0.5 (-1,2.5)	-2.0 (-2.5,2.5)	-2.5 (-4.5,3)	0.0 (-1.0,2.5)	0.0 (-2.5,2.5)	-2.2 (-4.5,3.0)
HFA z-score (range)	-0.4 (-1.9,3.6)	-0.57 (-2.83,8.02)	-3.1 (-7.6,7.5)	-0.12 (-4.5,3.6)	-0.69 (-6.5,5)	-0.7 (-7.6,8.0)
BMI-for-age z-score (range)	0.1 (-3.11,5.8)	-2.11 (-7.6,1.7)	-2.0 (-9.7,6.1)	0.5 (-0.1,3.5)	0.3 (-4.0,6.1)	-1.6 (-9.7,1.6)
TSF z-score (range)	-0.9 (-3.9,2.8)	-1.67 (-2.9,3.6)	-2.1 (-5.29,1.35)	-0.8 (-5.6,2.0)	-1.3 (-3.9,3.6)	-2.0 (-4.0,1.9)
MUAC z-score (range)	-0.9 (-5.1,6.5)	-2.04 (-5.5,6.5)	-3.08 (-5.5,1.54)	-0.9 (-5.1,6.5)	-1.1 (-5.5,6.5)	-2.7 (-5.1,1.5)

BMI=Body mass index;

HFA=Height-for-age;

MUAC=Mid-upper arm-circumference;

TSF=Tricipital skinfold; WFA=Weight-for-age

**Table 3.** Agreement between the STRONGkids malnutrition score classification and WHO classification

WHO class of malnutrition	n (%)	STRONGkids malnutrition risk			Modified STRONGkids		
		High	Medium	Low	High	Medium	Low
Severe	41 (15.1)	37	3	1	37	3	1
Moderate	59 (21.8)	29	28	2	26	31	2
No malnutrition	171 (63.1)	4	81	86	1	58	112
n (%)		70 (25.8%)	112 (41.3%)	89 (32.8%)	64 (23.7%)	92 (33.9%)	115 (42.4%)
Kappa	p=0.001		0.61			0.716	

According to the WHO classification of malnutrition, 100 children (37%) were malnourished, and of them, 41 (15%) had severe malnutrition. 48 (17.71%) had acute malnutrition (WFH z-score <-2 SD), of whom 17 (6.3%) were considered severe, and 37 (13.65%) had chronic malnutrition or stunting (HFA z-score <-2 SD), of whom 24 (8.9%) were considered severe. There were no significant differences in HFA z-score or WFH z-score between genders (1).

Using the STRONGkids screening tool, 115 (42.4%), 92 (33.9%) and 64 (23.7%) children had a low, moderate and high risk of malnutrition respectively. Those at high risk of malnutrition had lower median BMI, TSF, and MUAC.

Acute malnutrition (WFH z-score <-2 SD) was present in 89% of children with scores above 4, and in 10% of those with a score of 3. Chronic malnutrition (HFA z-score <-2 SD) was present in 16.2% of children with a score of 2, in 21.6% of those with a score of 3, and in 57% of those with scores above 4. Only 5.4% of those with score 0 or 1 had chronic malnutrition.

The kappa statistic for the correlation between STRONGkids and WHO malnutrition class was 0.61. When presence of a protein level of <6 mg/dL was used in upgrading the risk to the next category in order of severity of the STRONGkids tool assessment, the agreement between the STRONGkids and WHO malnutrition class improved significantly (kappa 0.716, p=0.001) (Table 3).

Smaller age and a longer duration of hospitalization were both associated with a higher malnutrition score (p=0.0001). As expected, children with underlying gastrointestinal disorders were most likely to have acute malnutrition (p=0.0051).

Children with normal nutritional status, who had a low STRONGkids score, had a significantly lower median BMI than those in the normal WHO category (0.7 vs 0.5 respectively) (p=0.0001); however, MUAC, TSF, and serum proteins did not differ significantly.

## DISCUSSION

Our study aimed to describe the prevalence of malnutrition in a cohort of hospitalized children at an academic hospital in Romania and to evaluate the ability of the STRONGkids screening tool to identify malnutrition risk in this population. In our study, the prevalence of malnutrition (37%) was significantly higher than that reported by others in both developed countries (5-7) and some developing countries, such as Iran (16). However, they were similar to reported rates in other developing countries, such as Turkey (8,9), with similar socioeconomic conditions as in Romania. Other developing countries, such as Thailand, reported much higher prevalence of malnutrition rates in their hospitalized children (50-60%) (21).

Children with a higher risk of malnutrition often have worse outcomes (22) and longer hospitalizations (16,23). While several nutritional risk assessment tools were developed in various paediatric inpatient populations, their usability cannot be generalized without a formal assessment.

In a cross-sectional study of 119 inpatient children and 100 children from the community, Moeeni *et al.* (16) evaluated the applicability of three nutritional risk assessment scores: the STRONGkids tool developed and validated in Netherlands (13), the Screening Tool for the Assessment of Malnutrition in Paediatrics (STAMP) (24), and the Paediatric Yorkhill Malnutrition Score (PYMS) (15). The latter two were developed and validated in the United Kingdom. While all three tools were easily administered (PYMS and STAMP by nurses and STRONGkids by physicians), neither were perfect. However, STRONGkids identified the most children at risk of malnutrition. Compared to data reported in their study, our cohort had a higher prevalence of underlying medical conditions (48.3% vs 25% respectively), and a higher proportion of children at high risk of malnutrition (23.7% vs 3.4%) while the overall population at risk of malnutrition were similar. These differences may be explained by the higher prevalence of children with underlying disease in our cohort, or specific socioeconomic differences not accounted for in either study.

In another study of 46 hospitalized children with inflammatory bowel disease, Wiskin *et al*. found that STRONGkids and STAMP tools are superior in assessing malnutrition risk compared to PYMS. However, the clinical relevance of these differences remains unclear (23).

In our cohort, the STRONGkids malnutrition risk score yielded a suboptimal agreement with WHO malnutrition class, likely due to socioeconomic, clinical, and demographic differences between our study and the original populations in which this score was developed. Importantly, the addition of an objective laboratory test (serum protein level) to the nutritional risk assessment, significantly improved the score's performance in our population. In contrast, the adjustment of cutoff points in the study of Moeeni *et al*. improved the PYMS malnutrition detection rates but this was not the case for STRONGkids or STAMP. (16)

### Limitations

Our study has several limitations. This is a single-centre experience and may not be representative of the whole Romanian paediatric population; thus, the results may not be extrapolated to other populations without further study. We chose a Caucasian, relatively uniform population from central Romania, without information whether the environment, geographical conditions, or eating habits might influence the results.

The test administration and anthropometric measurements were all performed by a single operator, and test reliability might have been improved by addition of another operator. The gold standard for the nutritional evaluation was WHO definition of malnutrition (17), and the objective data on body composition were not available. Last but not the least, the cross-sectional design of our study does not permit deriving causal inferences between malnutrition risk and length of hospital stay.

### Conclusions

A modified STRONGkids tool, incorporating serum protein levels, could be useful to identify hospitalized children at risk of malnutrition in a tertiary paediatric hospital in Romania. Further research is needed to evaluate whether nutritional risk assessments and targeted nutritional interventions help improve important patient-centred outcomes in hospitalized children.
